# Communication of poor prognosis between secondary and primary care: protocol for a systematic review with narrative synthesis

**DOI:** 10.1136/bmjopen-2021-055731

**Published:** 2021-12-22

**Authors:** Lucy V Pocock, Sarah Purdy, Stephen Barclay, Fliss E M Murtagh, Lucy E Selman

**Affiliations:** 1 Centre for Academic Primary Care, University of Bristol, Bristol, UK; 2 General Practice Research Unit, University of Cambridge, Cambridge, UK; 3 Wolfson Palliative Care Research Centre, Hull York Medical School, University of Hull, Hull, UK; 4 Population Health Sciences, University of Bristol, Bristol, UK

**Keywords:** primary care, palliative care, health informatics

## Abstract

**Introduction:**

People dying in Britain spend, on average, 3 weeks of their last year of life in hospital. Hospital discharge presents an opportunity for secondary care clinicians to communicate to general practitioners (GPs) which patients may have a poor prognosis. This would allow GPs to prioritise these patients for Advance Care Planning.

The objective of this study is to produce a critical overview of research on the communication of poor prognosis between secondary and primary care through a systematic review and narrative synthesis.

**Methods and analysis:**

We will search Medline, EMBASE, CINAHL and the Social Sciences Citation Index for all study types, published since 1 January 2000, and conduct reference-mining of systematic reviews and publications. Study quality will be assessed using the Mixed-Methods Appraisal Tool; a narrative synthesis will be undertaken to integrate and summarise findings.

**Ethics and dissemination:**

Approval by research ethics committee is not required since the review only includes published and publicly accessible data. Review findings will inform a qualitative study of the sharing of poor prognosis at hospital discharge. We will publish our findings in a peer-reviewed journal as per Preferred Reporting for Systematic review and Meta-analysis (PRISMA) 2020 guidance.

**PROSPERO registration:**

CRD42021236087

**Strengths and limitations of this study**

- This protocol conforms to the Preferred Reporting for Systematic review and Meta-analysis 2020 statement guidelines.
- As the concept of ‘poor prognosis’ is ill-defined in the literature, it is not possible to include it in the search strategy; instead proxies for poor prognosis will be used.
- We use a narrative synthesis approach as scoping searches suggest that a small number of studies will be identified, insufficiently similar in research design and context to enable meta-analysis.
- Narrative synthesis will provide an in-depth understanding of the literature on how poor prognosis is communicated between secondary and primary care clinicians, informing subsequent research.

## Introduction

In 2016, there were over 1.6 million emergency admissions for people in the last year of life in Britain, an average of three admissions per person.^1^ A study using Belgian GP data suggested that 60% of patients are hospitalised in the last 3 months of life, and in the USA, this was reported to be around 69%. These admissions can be traumatic for patients and their families, and could often be avoided.

A significant proportion of people die in the 12 months following an admission.^2 3^ Hospital admission is, therefore, a unique opportunity for hospital clinicians to identify patients with a poor prognosis (probably in the last year of life) and share this with general practitioners (GPs) at discharge. This can lead to improved continuity of care, providing a trigger for the GP to initiate Advance Care Planning (ACP) discussions with patients and their family/carers. The benefits of ACP remain uncertain, but are likely to go beyond the documentation of preferred place of care, level of treatment, and resuscitation status.^4^

Initiation of ACP with patients requires that GPs identify which patients are likely to be coming towards the end of their life and when to initiate discussions. This is challenging, particularly for patients with non-malignant disease, which often has a more unpredictable course than cancer.^5^ A recent systematic review highlighted that poor communication between professionals hindered the delivery of palliative care by GPs.^6^ A lack of communication from secondary care in relation to prognosis, patients’ previously expressed wishes and/or limitations to their treatment is a potential barrier to the initiation, or continuation, of ACP conversations by GPs: addressing these GP information needs would facilitate this process.^7 8^

Recent research shows that written forms of communication between secondary and primary care predominate, with information shared through referral letters, clinic letters and discharge summaries.^9^ Other studies have found that, although GPs would welcome telephone calls when a change in a patient’s status or care needs has occurred, secondary care clinicians report that getting through to GPs is problematic and time-consuming.^10 11^ Patient-held records are another potential solution. They are well established and effective in obstetrics and child health, but evaluations of patient-held records in cancer and palliative care have proved disappointing.^12–17^

Palliative care is often provided in parallel with active treatment. Consequently, there may not be discrete transition point in the type of care provided. Although the focus may shift from life-sustaining care to symptom management and quality of life, this may be not always be obvious to the patient and those involved in their care. This uncertainty makes communication at the primary/secondary care interface critical. Delays in receipt of a discharge summary have the potential to undermine GPs’ vital role in providing continuity of care.^10^ Asking patients to be responsible for delivering the discharge summary to the GP expedites this process, but may not always be appropriate or practical, particularly when patients are frail or at the end of life.^18^

Any recommendations about the communication of poor prognosis should be based on the best available evidence. To date, no comprehensive review has been completed in this area. This systematic review therefore aims to determine what processes are in use for communicating poor prognosis from secondary care to primary care. In accordance with the guidelines, our systematic review protocol was registered with the International Prospective Register of Systematic Reviews (PROSPERO) on 19 May 2021.^19^

## Aim

To determine what processes are in use for communicating poor prognosis from secondary care to primary care.

## Review questions

### PICO

**P**opulation—primary and secondary care clinicians, patients thought to be in the last year of life and their family/carers

**I**ntervention—any type of communication or intervention that enables secondary care clinicians to share information about a patient’s poor prognosis with primary care clinicians

**C**omparison—qualitative and mixed-methods studies may not have a comparison group; quantitative studies may compare the intervention with usual care or with a comparison/control group

**O**utcomes—facilitators of, and barriers to, the use of these interventions; acceptability and usefulness to patients, family/carers and clinicians; and impact on future care

#### Primary question

How is poor prognosis communicated between secondary and primary care?

#### Secondary questions

What are the facilitators of, and barriers to, this communication?

How acceptable and useful is this communication to patients, family/carers and clinicians?

What is the impact of this communication on patient care?

## Methods

### Inclusion criteria

Study reports will be included in this systematic review if they meet the following inclusion criteria:

1. Any study reporting original, empirical data, regardless of study design.
2. Studies published on, or after, 1 January 2000. This date has been chosen as a major reorganisation of the NHS. ‘The NHS Plan’^20^ was introduced in England in 2000 and models of care prior to this date are likely to be of less relevance to current practice. It was also when the Gold Standards Framework was introduced to improve care at the end of life.^21^
3. Studies reported in the English language, undertaken in any country.
4. Studies reporting any type of communication or intervention that facilitates the sharing of poor prognosis from secondary care to primary care. Examples include discharge summaries, clinic letters and shared electronic health records.
5. Studies reporting the views and experiences of:
  - Healthcare professionals working in secondary care (including mental health settings and acute hospitals) settings and/or in primary care (general practice).
  - Patients with incurable, advanced disease who have a poor prognosis (likely to be in the last year of life), regardless of age. Eligible conditions are described in table 1, although this list is not exhaustive. Under each heading in the table, common markers of poor prognosis for each condition are listed. Study reports will be included if they describe the population as having ‘advanced disease’, without further details, or if they describe markers of advanced disease, or poor prognosis, such as those listed in table 1, adapted from the Gold Standards Framework Proactive Identification Guidance, V.6.^22^
  - Current or bereaved carers of people with incurable, advanced disease, as defined above.

**Table 1:** Defining poor prognosis

*Cancer* Deteriorating performance status and functional ability due to metastatic cancer (stage IV disease), multi-morbidities. Cancer not amenable to treatment. Specific predictors of poor prognosis for cancer.	*Multiple sclerosis* Significant complex symptoms and medical complications. Dysphagia and poor nutritional status. Communication difficulties, for example, dysarthria and fatigue. Cognitive impairment.
*Heart disease* Coronary Heart Failure New York Heart Association stage 3 or 4 with ongoing symptoms despite optimal therapy. Repeated admissions with heart failure—three admissions in 6 months or a single admission aged over 75. Additional features include hyponatraemia <135 mmol/L, high BP, declining renal function and anaemia.	*Kidney disease* Stage 4 or 5 chronic kidney disease whose condition is deteriorating. Repeated unplanned admissions (more than 3 /year). Poor tolerance of dialysis with change of modality. Patients choosing the ‘no dialysis’ option (conservative), dialysis withdrawal or not opting for dialysis if transplant has failed. Symptomatic renal failure in patients who have chosen not to dialyse.
*Chronic obstructive pulmonary disease (COPD*) Recurrent hospital admissions (at least three in last year due to COPD). Medical Research Council (MRC) grade 4/5. Disease assessed to be very severe (eg, Forced Expiratory Volume in one second (FEV_1_) <30% predicted). Persistent symptoms despite optimal therapy. Too unwell for surgery or pulmonary rehabilitation. Fulfils long-term oxygen therapy criteria (PaO_2_ <7.3 kPa). Required ITU/NIV during hospital admission. Other factors, for example, right heart failure, anorexia, cachexia, >6 weeks steroids in preceding 6 months, requires palliative medication for breathlessness, still smoking.	*Stroke* Persistent vegetative, minimal conscious state or dense paralysis. Medical complications, or lack of improvement within 3 months of onset. Cognitive impairment/poststroke dementia. Other factors, for example, old age, male, heart disease, stroke subtype, hyperglycaemia, dementia, renal failure.
*Parkinson’s disease* Drug treatment less effective or increasingly complex regime of drug treatments. Reduced independence, needing help with activities of daily living (ADLs). Condition less well controlled with increasing ‘off’ periods. Dyskinesias, mobility problems and falls. psychiatric signs (depression, anxiety, hallucinations, psychosis).	*Frailty* Multiple morbidities. Deteriorating performance score. weakness, weight loss exhaustion. Slow walking speed.
*Motor neuron disease* Marked rapid decline in physical status. First episode of aspirational pneumonia. Increased cognitive difficulties. Weight loss. Significant complex symptoms and medical complications. Low vital capacity (below 70% predicted spirometry), or initiation of non-invasive ventilation. Mobility problems and falls. Communication difficulties.	*Dementia* Unable to walk without assistance. Urinary and faecal incontinence. No consistently meaningful conversation. Unable to do ADLs. Barthel score <3. Weight loss. Recurrent fever. Reduced oral intake. Aspiration pneumonia.

BP, blood pressure.

### Exclusion criteria

1. Studies not reported in English.
2. Studies published before 2000.
3. Studies reporting general communication from secondary care to primary care, without an emphasis on the sharing of poor prognosis.
4. Studies about patients who may have a poor prognosis, but where this has not been identified or communicated.
5. Studies reporting communication solely from primary care to secondary care, even if there is an emphasis on the sharing of poor prognosis.
6. Studies reporting the views and experiences of clinicians not working in secondary or primary care, or of patients, or carers of patients, who are not thought to be in the last year of life, including cancer survivors (people whose cancer is in remission and who are no longer being treated) and people with chronic but not life-limiting conditions, for example, diabetes, arthritis.
7. Case reports, protocols, editorials or commentaries.

Conference abstracts, letters and audits will be included if they present original empirical data and meet the other inclusion criteria. If there is uncertainty about whether the inclusion criteria are met, or if relevant data cannot be extracted from the study report, the authors will be contacted to ask if they can provide additional information and/or further data. If this is not possible the study will be excluded.

In the case of studies of mixed samples in which some, but not all, of the study population meets the inclusion criteria (eg, only a proportion of the patients in the study have advanced disease, or communication of poor prognosis from secondary to primary care is presented alongside other communication), if relevant data can be extracted it will be included. If relevant data cannot be extracted, the authors will be contacted to ask if they can provide relevant data; if this is not possible the study will be excluded.

### Search strategy

The following databases will be searched for English language studies:

Medline and EMBASE in Ovid, CINAHL and the Social Sciences Citation Index.

Additional hand searches of key journals, screening of reference lists of included studies, citation tracking and input from expert collaborators will supplement the database searches.

A search of the grey literature will be conducted through searches of key websites (eg, Marie Curie, King’s Fund, Department of Health), and relevant databases, such as OpenGrey and ProQuest, will also be searched. In addition, key authors of included studies will be contacted to see if further work has been completed.

Searches will be rerun prior to the final analysis to identify further recent studies for inclusion.

The Medline search strategy is shown in online supplemental appendix 1. This strategy will be adapted to the other electronic databases, with any modifications reported in the review manuscript.

10.1136/bmjopen-2021-055731.supp1 Supplementary data

Poor prognosis is not included within the search strategy, as it is difficult to define and attempts to do so result in a less sensitive search, that is, potentially relevant papers are missed.

Databases searches were run on 17 May 2021. The expected end date for the review is 30 November 2021.

### Screening and data extraction

Search results from each database will be downloaded and managed in Covidence, an online review management platform.^23^ Each title and abstract will be screened against the inclusion/exclusion criteria by the lead reviewer (LVP). A second reviewer will independently screen a sample of 25% of the titles and abstracts. Any differences of opinion will be discussed and, if necessary, adjudicated by a third reviewer. Full-text screening will then be conducted by LVP.

LVP will extract data from the included studies using a prepiloted data extraction template in Covidence. Data extraction will be checked by a second reviewer and modified where needed. Empirical data from the results sections of each paper that are pertinent to the review questions will be recorded in the data-extraction form and entered into NVivo for synthesis. Missing data will be requested from study authors via email.

### Quality assessment

The quality of all included studies will be assessed independently by two reviewers using the Mixed-Methods Appraisal Tool (MMAT), V.2018.^24^ This validated tool is appropriate for this review as it can be applied to qualitative, quantitative (randomised, non-randomised and descriptive) and mixed-methods study designs. The tool uses a set of questions specific to study design, converted into four possible scores (worst to best: 25/50/75/100). Disagreements between the reviewers will be resolved through discussion, involving a third reviewer if necessary. No studies will be excluded on the basis of their quality, but the narrative synthesis will reflect on the quality of the identified studies.

### Evidence synthesis

We will conduct a narrative synthesis of the studies, following the framework stages proposed by Popay *et al*,^25^ adapting these where necessary:

1. Developing a theoretical model of how different forms of communication work, why and for whom.
2. Developing a preliminary synthesis.
3. Exploring relationships in the data.
4. Assessing the robustness of the synthesis product.

The narrative synthesis will be structured around the strategies for communication of poor prognosis, the facilitators of, and barriers to, this communication, the evidence of the acceptability to patients, family/carers and clinicians and the impact of this communication on patient care.

### Patient and public involvement

The research question was developed at a series of workshops attended by members of the University of Bristol Palliative and End of Life Care Research Group Patient and Public Involvement (PPI) Advisory Panel. The PPI Advisory Panel did not contribute to the design of the systematic review, but will be consulted on the dissemination of results and how this feeds into later work in this subject area.

## Ethics and dissemination

This systematic review of published/publicly available studies is exempt from ethical approval. The review will be reported as per Preferred Reporting for Systematic review and Meta-analysis guidance^26^ and published in a peer-reviewed journal. The findings will inform a qualitative study to investigate the communication of poor prognosis from secondary care to primary care at the point of hospital discharge.
