# Supplementary material for: Communication of poor prognosis between secondary and primary care: protocol for a systematic review with narrative synthesis
Source: BMJ Open. 2021 Dec 22;11(12):e055731. doi: 10.1136/bmjopen-2021-055731 (PMC9066345; doi:10.1136/bmjopen-2021-055731)
Supplement: Supplementary data [file bmjopen-2021-055731supp001.pdf]

Appendix 1 – Medline search strategy

1. Communication/
2. communicat*.tw.
3. interprofessional relations/
4. interdisciplinary communication/
5. Interprofessional Relat*.tw.
6. Inter-professional relat*.tw.
7. interprofessional collaborat*.tw.
8. information transfer.tw.
9. electronic health records/
10. health information exchange/
11. Electronic Health Record*.tw.
12. ninformation exchange.tw.
13. "Continuity of Patient Care"/
14. discharge summar*.tw.
15. electronic palliative care coordination system*.tw.
16. health communication/ or correspondence as topic/ or electronic mail/
17. (clinic letter* or poor prognosis letter).tw.
18. handover*.tw.
19. 1 or 2 or 3 or 4 or 5 or 6 or 7 or 8 or 9 or 10 or 11 or 12 or 13 or 14 or 15 or 16 or 17 or 18
20. general practitioners/
21. physicians, family/
22. physicians, primary care/
23. general practi*.tw.
24. Family practi*.tw.
25. family physician.tw.
26. Primary Health Care/
27. primary health care.tw.
28. primary team.tw.
29. primary care.tw.
30. 20 or 21 or 22 or 23 or 24 or 25 or 26 or 27 or 28 or 29
31. medical staff, hospital/
32. hospitalists/
33. hospital physician.tw.
34. Secondary Care/
35. secondary care.tw.
36. (oncolog* or cancer).tw.
37. hospital medicine/
38. cardiology/
39. cardiolog*.tw
40. gastroenterology/
41. hepatolog*.tw.
42. hematology/
43. medical oncology/
44. nephrology/
45. pulmonary medicine/

46. respiratory physician.tw
47. neurology/
48. palliative medicine/
49. Palliative Care/
50. Terminal Care/
51. Frailty/
52. geriatrics/
53. geriatric*.tw.
54. internal medicine/
55. specialties, surgical/
56. colorectal surgery/
57. general surgery/
58. gynecology/
59. neurosurgery/
60. surgical oncology/
61. thoracic surgery/
62. urology/
63. (surgery or surgeon).tw
64. 31 or 32 or 33 or 34 or 35 or 36 or 37 or 38 or 39 or 40 or 41 or 42 or 43 or 44 or 45 or 46 or 47 or 48 or 49 or 50 or 51 or 52 or 53 or 54 or 55 or 56 or 57 or 58 or 59 or 60 or 61 or 62 or 63
65. "primary/secondary interface".tw
66. "between care settings".tw
67. hospital to primary care.tw
68. 65 or 66 or 67
69. 19 and ((30 and 64) or 68)
